# Influence of Storage Time and Method of Smoking on the Content of EPA and DHA Acids and Lipid Quality of Atlantic Salmon (*Salmo salar*) Meat

**DOI:** 10.1155/2022/1218347

**Published:** 2022-02-23

**Authors:** Grzegorz Bienkiewicz, Grzegorz Tokarczyk, Patrycja Biernacka

**Affiliations:** ^1^Department of Commodity Science, Quality Assessment, Process Engineering and Human Nutrition, West Pomeranian University of Technology, Papieża Pawła VI 3, 71-459 Szczecin, Poland; ^2^Department of Fish, Plant and Gastronomy Technology, West Pomeranian University of Technology, Papieża Pawła VI 3, 71-459 Szczecin, Poland

## Abstract

Smoking is one of the oldest technologies for processing and preserving raw materials of animal origin. To this day, smoked fish is very popular among consumers. The most popular smoked fish is salmon. The research compared the qualitative changes in the fat fractions of hot and cold-smoked salmon during refrigerated storage. Generally accepted physicochemical methods for assessing the quality of fats, such as peroxide, anisidine, and acid number, were used. First, the smoked salmon was stored, and then, the samples were analyzed to find changes in the fatty acids EPA (eicosapentaenoic acid) and DHA (docosahexaenoic acid). It was shown that cold smoking significantly inhibited the increase in the level of lipid oxidation compared to hot smoking and raw samples stored in the same way. In the meat of stored and cold- and hot-smoked salmon, the TOTOX values remained at the level indicated by the Codex Alimentarius. Hot smoking limited the degree of lipid hydrolysis during storage as compared to cold smoking. The smoking process had a protective effect on EPA and DHA acids. In the raw samples, the loss of these acids was three times higher. Summarizing the research, it can be concluded that smoked products are a good and safe source of omega-3 fatty acids in the diet.

## 1. Introduction

Aquaculture is a leading role in meeting the growing demand for fish and seafood, as well as increasing sustainable fisheries, which has a significant impact on the environment. The production of fish from aquaculture for food purposes in the European Union in 2018 was over 1.3 million tonnes by weight of live fish [1]. Salmonidae is one of the most economically important aquaculture species in the world. Atlantic salmon (*Salmo salar*) ranks first among global Salmonidae with an annual production of more than two million tons [2].

Poland is the largest producer of smoked fish in Europe. In 2019, a total of over 82,000 tonnes of smoked fish were produced in Poland. The main species for smoking were salmon, mackerel, and trout. The salmon totalled over 57,000 tons [3].

Smoking, drying, marinating, and salting fish are the most traditional methods of preservation [4]. The smoking process is one of the oldest preservation methods, extending the shelf-life and adding flavour and aroma to food. Such products can be smoked cold or hot. Salmon is the most popular cold-smoked fish and retains its raw, translucent, dark pink colour. Hot-smoked salmon takes on a pale pink and opaque appearance. During hot smoking, food is cooked and smoked at the same time, and the taste is created as a result of the action of smoke and the processes of glycation and lipid degradation [5, 6].

Therefore, smoke can perform a protective function increasing the durability and safety of food and smoking as a technological treatment camouflaging the defects and bad quality of the raw material [7]. In the article by Popelka et al. [8], the influence of smoking and packaging methods was determined, the physicochemical and microbiological quality of smoked mackerel (*Scomber scombrus*). Research shows that the smoking process, as well as appropriate packaging, can protect lipids, including polyunsaturated fatty acids, against oxidation.

The study assumes that the type of smoking of the fish will have a significant impact on the qualitative changes in the lipid fraction and losses of EPA and DHA acids during the refrigerated storage of the finished product. Atlantic salmon were selected for the research, as the species most popular on the European market, consumed both cold and hot-smoked. Because salmon fish and especially Atlantic salmon are rich in protein and essential polyunsaturated fatty acids (PUFAs) such as docosahexaenoic acid (DHA), eicosapentaenoic acid (EPA), and arachidonic acid (ARA), demand for these fishes is increasing [9, 10].

To maintain the health-promoting effect of fish lipids, it is important to ensure their high quality. So far, most research on the quality of smoked fish products has focused on the degree of freshness, sensory values, and microbiological quality related to their safety. Nevertheless, it must not forget about the quality of lipids and changes in their degree of freshness during technological processes [11], as well as during storage related to the distribution and shelf life of the finished product.

The purpose of the research was to compare the changes in the oxidative lipids of salmon (*Salmo salar*) meat occurring during cold and hot smoking and during the storage of the refrigerated finished product.

## 2. Materials and Methods

### 2.1. Raw Material

The study was carried out on farmed fish Atlantic salmon (*Salmo salar*) obtained from an aquaculture facility “Jurassic Salmon,” located in West Pomeranian province in Poland. All fish (15 individuals for each type of smoking) were harvested in June 2021, minimizing quality differences between the raw materials. The fish were 80-90 cm in length, and the average weight of these fish was 2.7 kg ± 100 g. Individuals were slaughtered in the fish farm; the gills were cut off and then bled in a water-ice mixture. After that, the fishes were packaged in expanded polyester boxes with ice and transported to the laboratory during 12 h under crushed ice condition. Fish-to-ice ratio was 1 : 1. The fish were beheaded, gutted, and washed. Then, the carcasses were filleting, and fillets obtained were washed again with water at 4°C. After salting from each fillet, two pieces were cut from the central part of it (length—10 cm).

### 2.2. Sampling

Samples were taken on the arrival of the raw material, after smoking and after 1, 2, 3, and 4 weeks of storage for the evaluation of peroxide value (PV), anisidine value (AsV), and acid value (AV). Additionally, lipid content, water content, and total volatile basic nitrogen (TVB-N) were measured and water activity (aw) was determined.

### 2.3. Processing

#### 2.3.1. Salting

The same brine salting techniques were used for the different smoking techniques [fish-to-brine ratio was 1 : 1 (*w*/*v*)]. Brine was prepared by dissolving 200 g refined NaCl (minimum 99.8% NaCl, Kopalnia Soli “KŁODAWA” S.A., Poland) per 1000 ml of water (8-10°C). The brine was vigorously stirred for 1 h and left to stabilise the temperature (14-15°C) and to ensure complete dissolution of the salt. Fillets for cold smoking were brine salted for 3 hours to achieve about 4.0% salt concentration in the salmon muscle. For hot smoking, fillets were brine salted for 45 min to achieve about 2.0% salt concentration in the muscle. Afterwards, the fillets were rinsed briefly with water (15°C) in order to remove the excess of NaCl and stored at 14-15°C for 30 min until smoking.

#### 2.3.2. Smoking

*(1) Cold Smoking*. The cold smoking process of the fillets was carried out using a KGW-50-E smoking cabinet (STAWIANY, Pszczółki, Poland) equipped with a INDU iMAX 500 KW-V Microprocessor (MIKSTER Sp. Z o.o., Czeladź, Poland) and a smoke generator with automatic ignition and dosing. Alder wood chips (AWPOL, sp. k., Wierzchosławice, Poland) were moistened (100 ml water/kg chips) and used for smoke generation by combustion of the chips. The fillets were placed at the trolley grids before processing.

After storage for 30 min at 14-15°C, the smoking process began with a drying step in the smoking oven for 180 min at 20°C, followed by a smoking step at 20°C ± 1°C for 300 min. The relative humidity was set to 65%, and air velocity was 2.0 ms^−1^.

*(2) Hot Smoking*. Fish were smoked in the same smoking cabinet as used before for the cold smoking, using the same alder wood chips. The smoking process consisted of three steps:

(Step 1) Drying: the fish were dried for 60 minutes at 30-40°C with high air circulation.
(Step 2) Smoking: smoking was performed for 90 minutes at 40-65°C. The temperature increased proportionally with time.
(Step 3) Heating: the temperature of the smoking chamber was increased to 75°C for 40 minutes. This allowed to obtain 63°C in the core of the fish.

#### 2.3.3. Packaging

After cold smoking and each stage of hot smoking, fish were stored at 14-15°C for 30 min and then were cooled to a temperature of 3°C or below within 12 hours. Chilled fish were vacuum packaged (Webomatic Machinenfabrik GmbH, Bochum, Germany) and then stored in a cooled room at +6°C until analysis.

### 2.4. Chemical and Physical Analyses

#### 2.4.1. Determination of Lipid Content (Smedes Method)

Lipids were extracted by cyclohexane and propan-2-ol and transferred to the cyclohexane phase by the addition of water. Phase separation was performed by centrifugation. Gravimetric fat was determined after separation of the cyclohexane layer and evaporation to dryness [12].

#### 2.4.2. Determination of Moisture, Water Activity, Total Volatile Bases Nitrogen, and Salt Content

Moisture was determined gravimetrically after drying the material in an oven at 105°C according to the AOAC [13] method.

Water activity (aw) was determined in triplicate using the HygroLab C1 instrument (Rotronic, Switzerland), equipped with a HC2-AW probe, calibrated in the range 0.1-0.95 with solutions of LiCl of known activity [14].

Total volatile bases nitrogen (TVB-N) was determined by Conway and Byrne [15] method according to the Commission Regulation (EC) No. 2074/2005 of 5 December 2005 [16].

The salt is extracted by water from the preweighed sample. After the precipitation of the proteins, the chloride concentration is determined by titration of an aliquot of the solution with a standardized silver nitrate solution (Mohr method) and calculated as sodium chloride [17].

### 2.5. Quality Analysis of Lipids

Lipids were extracted using the Bligh and Dyer [18] method. Single-phase lipid solubilization with a chloroform-methanol mixture (1 : 1) was used. Quantification results were expressed as grams of lipid per 100 g of muscle tissue.

#### 2.5.1. Determination of Lipid Quality Parameters

Peroxide value (PV) was determined in the lipid extract by peroxide reduction with ferric thiocyanate, according to Pietrzyk [19] based on the oxidation of ferrous salt by hydroperoxides and the reaction of ferric salts with potassium isothiocyanate. The red ferric complexes formed were determined spectrophotometrically. Results were expressed as milliequivalents of oxygen per kilogram of lipids (meqO_2_/kg of lipids).

Anisidine value (AsV) was determined in fish muscle according to the AOCS [20] method, based on the reaction between *α*- and *β*-unsaturated aldehydes (primarily 2-alkenals) and p-anisidine reagent. AsV was expressed as 100 times the absorbance measured at 350 nm (Thermo Scientific, Genesys 20) in a 1 cm path length cuvette from a solution containing 10 mg of lipid in 1 ml of reaction medium.

Acid value (AV) was determined by titration of 0.1 N KOH in methanol, according to Polish Standard method [21]. Results were expressed as percent of free fatty acid calculated as oleic acid (% FFA).

### 2.6. Determination of Fatty Acids

Fatty acid methyl esters (FAME) were obtained from the tissue by alkaline hydrolysis of extract of lipids with 0.5 N sodium methylate (CH_3_ONa) [22]. Next, the FAMEs were separated using a gas chromatography apparatus, coupled with a mass spectrometer (Agillent Technologies 7890A), and equipped with a split/splitless type injector. Conditions of FAME separation were as follows: column SPTM 2560, 100 m 0.25 mm ID, 0.20 lm film, catalogue no. 24056; carrier gas: helium at a constant flow rate of 1.2 ml/min; split 1: 50; injector temperature: 220°C; detector temperature: 220°C; programmed furnace temperature: 140°C (5 min) increased to 240°C at a rate of 4°C/min; analysis time: 45 min. The qualitative interpretation of chromatograms was based on the comparison of retention times and mass spectra of the particular FAMEs of the sample with those of analogous FAME standards by Sigma Company (Lipid Standard). As an internal standard, C 19:0 was used.

### 2.7. Statistical Analysis

The results were consumed in triplicate and then averaged. Statistical analysis was based on one-way analysis of variance, homogeneous groups were created by Duncan's test for *P* ≤ 0.05. The data was statistically analyzed using Statistica 13.0 StatSoft Inc. data analysis software.

## 3. Results and Discussion

### 3.1. Changes in the Amount of Fat during Storage

**Figure 1:**
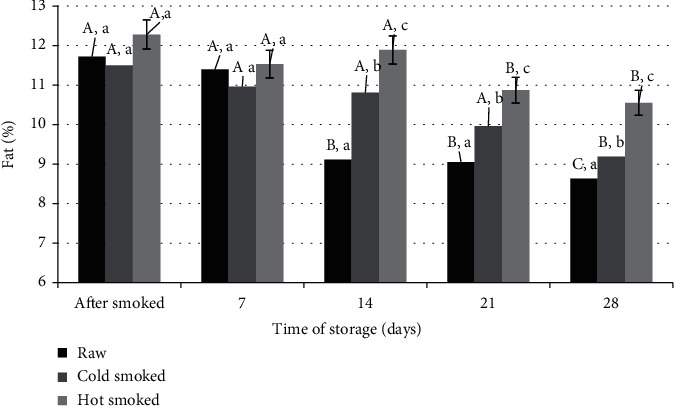
Comparison of changes in fat content during storage of raw, cold-smoked, and hot-smoked salmon. a, b, c: significant differences (*n* = 3) between individual treatments within one storage time. A, B, C: significant differences (*n* = 3) between processing methods during storage.

The starting fat content of raw salmon fillets averaged 11.72%. Immediately after smoking, the fat content was 11.50% for cold-smoked fish and 12.28% for hot-smoked fish. Similar results of fat content during salmon smoking were also reported by Robb et al. [23]. However, these differences were not statistically significant, despite various technological treatments during smoking (Figure 1). During the refrigerated storage at 6°C of the vacuum-packed samples, a decrease in the fat content was observed depending on the storage time and the form of smoking used. After 28 days of storage, the greatest losses of fat content were recorded in the raw samples. The fat content in the meat decreased to 8.63%. On the other hand, the lowest fat content was in hot-smoked samples (10.55%).

### 3.2. Determination of Moisture, Water Activity, Total Volatile Bases Nitrogen, and Salt Content

These changes were mainly caused by the leakage which also affected the water content (Table 1). The higher fat content in the hot-smoked samples could have been influenced by their better extractability compared to the raw and cold-smoked samples. At the heating stage, during the hot smoking process, apart from the loss of water, proteins are denatured, and thus tissue lipids are more easily extracted [24, 25].

It was indirectly shown by Bienkiewicz et al. [26] by examining the quality of the extracted fat at the various stages of trout smoking. The authors found differences in the extractability of fat between raw and postheating samples using selective extraction methods.

### 3.3. Changes in the Quality Parameters of Fat during Storage Depending on the Type of Smoking

In order to characterize the qualitative changes in the lipid fraction of raw and smoked salmon stored under refrigeration conditions, the amount of peroxides (PV), secondary oxidation products (AsV), and the degree of lipid hydrolysis (AV) were determined.

#### 3.3.1. Oxidative Changes

**Figure 2:**
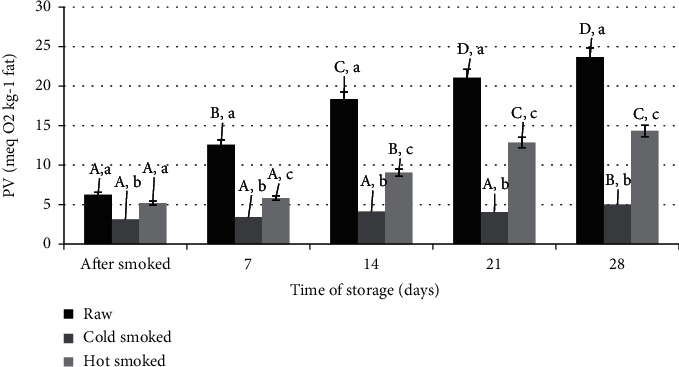
Comparison of changes in the peroxide number during storage of raw, cold- and hot-smoked salmon. a, b, c: significant differences (*n* = 3) between individual treatments within one storage time. A, B, C, D: significant differences (*n* = 3) between treatments during storage.

Figure 2 shows the changes in the primary oxidation products expressed as PV. Comparing the influence of the smoking process on the level of oxidation, it was found that the lipids of cold-smoked salmon were characterized by the lowest content of peroxides. Raw salmon had an initial PV of 6.25 meqO_2_/kg fat, similar to that of hot-smoked salmon lipids, while cold-smoked salmon lipids contained half as much peroxide. The smoking step radically reduced the amount of peroxides, below the level at the start of the process. Such a reduction of primary oxidation products may be caused by saturation of fat with components of smoke, which has strong antioxidant properties [5, 26, 27]. Similar changes were also observed during refrigerated storage. The lipid oxidation levels of the cold-smoked salmon were very stable. Only on the 28th day, a statistically significant increase in lipid oxidation was observed, but still minor compared to the lipids from raw and hot-smoked samples. In this case, an additional catalyst the autooxidation processes could be the heating step in the smoking process, in which no smoke was used. Research by Domiszewski [28] has shown that heating the muscle tissue of trout, herring, and sprat at a temperature of 60-80°C catalyses autooxidation processes. Raw salmon lipid oxidation increased with storage time up to 23.66 meqO_2_/kg fat. In the case of oily fish, the phenomenon of dynamic autooxidation during storage is widely documented [29, 30].

**Figure 3:**
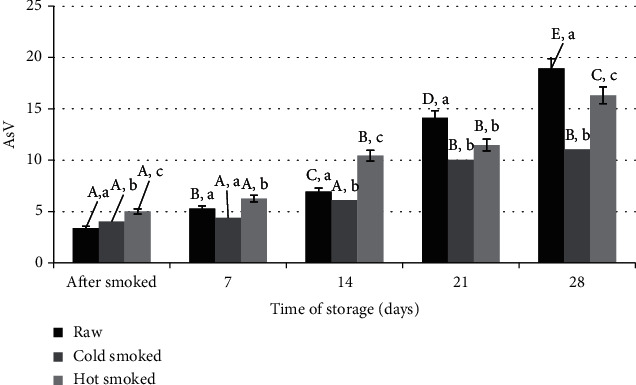
Comparison of changes in anisidine number during storage of raw, cold-smoked, and hot-smoked salmon. a, b, c: significant differences (*n* = 3) between individual treatments within one storage time. A, B, C, D, E: significant differences (*n* = 3) between treatments during storage.

The amount of secondary oxidation products, expressed as an anisidine number (AsV), ranged from 3.40 for raw salmon to 5.01 for the hot smoked sample (Figure 3). It was a slight but statistically significant increase, which could be caused by the heating stage up to 75°C in the smoking process [28]. Along with the storage time, an increase in secondary oxidation products was noted which was the most dynamic for the raw and hot-smoked samples. On day 28 of refrigeration, the anisidine numbers were 18.94 and 16.32, respectively. The formation of aldehydes in fish can be attributed to enzymatic reactions, but in the case of the hot smoking process, it can be mainly attributed to thermal degradation, Maillard reactions, and lipid changes. These reactions take place during thermal processes such as heating, baking, or smoking [31–33].

An anisidine number, as well as TBARS tests, is used to characterize qualitative changes in aldehydes and ketones. Analyzing the literature data, the use of the TBARS method for the determination of secondary oxidation products in fish fat was observed [34, 35]. The use of the test of expressed AsV allows the determination of the TOTOX index, which characterizes the overall level of oxidation. This index has been used to refer to the limits for oxidation of fish oils as defined in the Codex Alimentarius [36]. TOTOX ≤ 26 was adopted as the acceptable value. Calculated on the basis of the equation 2xPV + AsV. Comparing the obtained results, it was found that the lipids of the samples stored under refrigerated conditions exceeded this level for raw salmon after 14 days and for hot-smoked salmon after 21 days. The lipids of cold-smoked salmon on day 28 had a TOTOX value of 21.09, so it was lower than assumed in the Codex Alimentarius standards.

#### 3.3.2. Hydrolytic Changes

**Figure 4:**
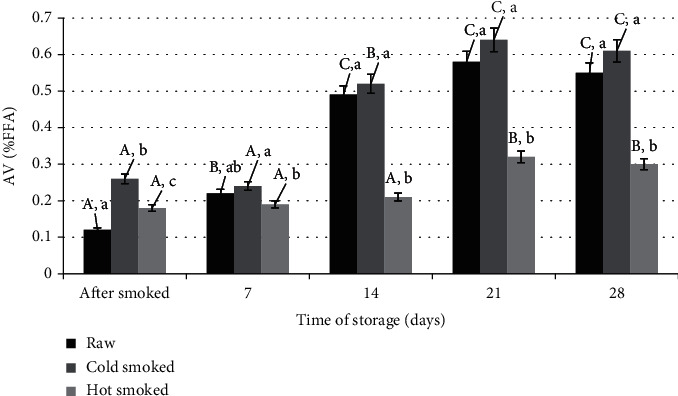
Comparison of changes in acid number during storage of raw, cold-smoked, and hot-smoked salmon. a, b, c: significant differences (*n* = 3) between individual treatments within one storage time. A, B, C: significant differences (*n* = 3) between treatments during storage.

The increase in the amount of FFA in the smoking process as compared to the raw salmon samples was visible immediately after the completion of the process (Figure 4). The same dependence was found by Tokarczyk et al. [11] by analysing the changes in the amount of FFA during hot smoking of whitefish. The amount of FFA, expressed as acid number (AV), further increased with the storage time. It is particularly clearly visible after the 14th day of storage, where in the raw and cold-smoked samples the amount of FFA is almost twice as high as in the hot-smoked samples. On the one hand, it may be related to the greater availability of water catalysing lipolytic processes and the higher water activity of these products (Table 1). Changes in water activity and oxidative processes during storage of raw fish material are reported by Giannakourou et al. [37]. In this study, minnow technology was used to extend the shelf life of European eel (*Anguilla anguilla*) fillets. It was found that the use of a traditional food preservation method such as smoking reduces lipid oxidation processes with storage time, but it is still necessary to search for methods to minimize these processes, also associated with high water activity [38]. Such relationships were observed in their study, except that on raw meat, by Zhou and Zhao [39] and Huang et al. [40]. They found that the parameters of technological process have a significant influence on the course of hydrolytic processes. They affect changes in water activity, which can interfere with lipolytic activity.

### 3.4. Changes in the Content of EPA and DHA Acids

From a nutritional point of view, omega-3 fatty acids are a particularly valuable food ingredient. All dietary recommendations indicate the need to increase the omega-3 long-chain fatty acids (LC n-3 PUFA) in the diet [41]. Fish, depending on the species and breeding method, is one of the best sources of EPA and DHA. However, their high degree of unsaturation and the lack of natural antioxidant substances in fish tissue [42, 43] makes that many technological processes may lead to their oxidation and quantitative losses. Therefore, it is important to optimize the technological processes of fish processing so as to protect fish lipids as much as possible against factors catalysing their oxidation.  Table 2 presents changes in the content of the sum of EPA and DHA acids during the cold storage process of raw, cold-smoked, and hot-smoked salmon.

According to the presented data, salmon is a good source of omega-3 fatty acids. Consuming an average fat content of over 10% (Figure 1), only 50 g of salmon covers the recommended daily intake of omega-3 fatty acids [44, 45]. The output content of the sum of these acids was 152 mg/g fat for raw salmon. These amounts are consistent with literature data; however, they may vary depending on the season and type of breeding [46].

During the smoking process, both hot and cold, a decrease in the content of EPA and DHA acids was observed, especially significantly in the case of hot smoking. A reduction in lipid content, especially EPA and DHA, was observed by Domiszewski and Mierzejewska [47] studying the effect of processing on the actual retention of eicosapentaenoic and docosahexaenoic acid, lipid oxidation, and physical properties of canned smoked sprat (*Sprattus sprattus*). The kinetics of lipid quality changes in salmon during thermal processing was also described by Kong et al. [48]. Domiszewski [28] conducted a study that showed a decrease in the percentage of EPA and DHA by about 10% when heating fish up to 60 minutes. Heating fish at higher temperatures and longer times can cause a decrease in EPA and DHA content to about 20-25%.

Storage for 28 days resulted in further, but less significant, decreases in these acids. The greatest losses were associated with raw samples. Here, the loss was 30 mg/g of fat, while in smoked samples, it was almost three times less. Undoubtedly, the protection of omega-3 acids during storage was influenced by smoke [26], which has strong antioxidant properties as shown in Section 3.1.

## 4. Conclusion

Nowadays, more and more fish raw materials come from aquaculture as it is becoming harder and harder to find high-quality fish raw materials from ocean fisheries. A common example is Atlantic salmon, which for processing is mainly delivered from Norwegian, Icelandic, or Canadian farms. It is a species that is mainly smoked and intended for trade in the form of chilled portions packed in vacuum or in skin technology. Therefore, it is important to maintain the high quality and safety of this type of products for as long as possible. The available literature contains a lot of information on microbiological safety or freshness level indicators, such as TVB-N. This experiment showed that the 28-day storage period of the hot and cold smoked product did not result in exceeding the TVB-N limits. However, in the case of raw salmon, on day 21 of storage, a TVB-N content limit was found.

The present researcher compared the effect of salmon smoking method on the quality changes of lipid fraction and the effect of refrigerated storage of vacuum-packed hot- and cold-smoked salmon fillets. It has been shown that smoking, especially cold smoking, lowers the level of lipid oxidation and significantly inhibits autooxidation processes during storage. Hot smoking, on the other hand, reduces hydrolytic changes in fat better than cold smoking. Limiting the dynamics of autooxidation during refrigerated storage of smoked salmon fillets significantly reduces the loss of nutritionally valuable EPA and DHA acids. Storage of raw samples resulted in three times greater losses of these acids. Another significant problem, which, admittedly beyond the scope of this work, seems to be very important for technological and qualitative reasons, is the leakage generated during storage, which affects the overall fat content.

## Figures and Tables

**Table 1:** Changes in physicochemical parameters in raw salmon after cold and hot smoking.

Time (days)	Salt (%)	Water (%)	Water activity	TVB-N (mg/100 g)
Raw fish				
Raw	nd	68.92 ± 1.89	0.994	2.11 ± 0.05
7	nd	66.45 ± 2.12	0.993	8.22 ± 0.09
14	nd	66.11 ± 1.02	0.994	17.48 ± 0.35
21	nd	65.21 ± 0.69	0.992	24.96 ± 0.32
28	nd	63.14 ± 2.54	0.989	45.16 ± 1.35
Cold-smoked				
After smoked	4.25 ± 0.15	63.48 ± 2.25	0.975	1.54 ± 0.35
7	4.24 ± 0.09	62.59 ± 1.05	0.977	2.66 ± 0.65
14	4.01 ± 0.08	61.11 ± 1.65	0.978	3.96 ± 0.75
21	4.05 ± 0.11	60.98 ± 1.09	0.965	8.35 ± 0.95
28	4.01 ± 0.12	60.14 ± 2.01	0.961	9.87 ± 0.45
Hot-smoked				
After smoked	2.02 ± 0.25	58.19 ± 0.94	0.978	1.12 ± 0.45
7	2.04 ± 0.15	58.08 ± 2.01	0.969	3.11 ± 0.65
14	2.01 ± 0.04	56.89 ± 1.11	0.966	3.68 ± 1.01
21	2.05 ± 0.07	56.09 ± 0.75	0.968	5.28 ± 1.12
28	2.11 ± 0.09	56.14 ± 0.85	0.969	12.82 ± 2.03

**Table 2:** Changes in the sum of EPA and DHA acids (mg/1 g fat) of raw, cold-smoked, and hot-smoked salmon, stored for 28 days in refrigerated conditions.

EPA + DHA (mg/1 g of fat)					
	After smoking	7	14	21	28
Raw	152 ± 9.1^A,a^	139 ± 1.2^B,a^	133 ± 1.17^B,a^	128 ± 8.2^C,a,b^	122 ± 3.6^C,a,b^
Cold-smoked	148 ± 1.1^A,a^	140 ± 1.0^B,a^	138 ± 2.5^B,a^	139 ± 3.9^B,a^	137 ± 4.1^B,c^
Hot-smoked	124 ± 1.5^A,b^	115 ± 1.9^B,b^	116 ± 9.1^B,b^	117 ± 11.5^B,b^	118 ± 8.5^B,b^

a, b, c: significant differences (*n* = 3) between individual treatments within one storage time; A, B, C: significant differences (*n* = 3) between treatments during storage.

## Data Availability

The data used to support the findings of this study are available from the corresponding author upon request.
